# Circulating Tumor Cells and Circulating Tumor DNA Detection in Potentially Resectable Metastatic Colorectal Cancer: A Prospective Ancillary Study to the Unicancer Prodige-14 Trial

**DOI:** 10.3390/cells8060516

**Published:** 2019-05-28

**Authors:** François-Clément Bidard, Nicolas Kiavue, Marc Ychou, Luc Cabel, Marc-Henri Stern, Jordan Madic, Adrien Saliou, Aurore Rampanou, Charles Decraene, Olivier Bouché, Michel Rivoire, François Ghiringhelli, Eric Francois, Rosine Guimbaud, Laurent Mineur, Faiza Khemissa-Akouz, Thibault Mazard, Driffa Moussata, Charlotte Proudhon, Jean-Yves Pierga, Trevor Stanbury, Simon Thézenas, Pascale Mariani

**Affiliations:** 1 Department of Medical Oncology, Institut Curie, PSL Research University, 75005 Paris, France; francois-clement.bidard@curie.fr (F.-C.B.); luc.cabel@curie.fr (L.C.); jean-yves.pierga@curie.fr (J.-Y.P.); 2 Circulating Tumor Biomarkers Laboratory, Institut Curie, PSL Research University, 75005 Paris, France; jordanmadic@yahoo.fr (J.M.); saliou.adrien@gmail.com (A.S.); aurore.rampanou@curie.fr (A.R.); charles.decraene@curie.fr (C.D.); charlotte.proudhon@curie.fr (C.P.); 3 UVSQ, Paris Saclay University, 92210 Saint Cloud, France; 4 Department of Digestive Oncology, ICM Regional Cancer Institute of Montpellier, 34298 Montpellier, France; marc.ychou@icm.unicancer.fr (M.Y.); t-mazard@chu-montpellier.fr (T.M.); 5 Department of Oncology, Montpellier University, 34000 Montpellier, France; 6 INSERM U830, Institut Curie, PSL Research University, 75005 Paris, France; marc-henri.stern@curie.fr; 7 CNRS UMR144, Institut Curie, PSL Research University, 75005 Paris, France; 8 Department of Medical Oncology, Hôpital Robert Debré, Reims University Hospital, 51100 Reims, France; obouche@chu-reims.fr; 9 Department of Digestive Oncology, Centre Léon Bérard, 69008 Lyon, France; michel.rivoire@lyon.unicancer.fr; 10 INSERM U866, Centre Georges-François Leclerc, 21000 Dijon, France; fghiringhelli@cgfl.fr; 11 Department of Medical Oncology, Centre Antoine Lacassagne, 06189 Nice, France; eric.francois@nice.unicancer.fr; 12 Department of Digestive Oncology, CHU de Toulouse, 31059 Toulouse, France; guimbaud.r@chu-toulouse.fr; 13 Department of Digestive Oncology, Institut Sainte Catherine, 84000 Avignon, France; l.mineur@isc84.org; 14 Department of Gastroenterology, Hôpital Saint Jean, 66000 Perpignan, France; faiza.khemissa@ch-perpignan.fr; 15 Department of Gastroenterology, CHRU de Tours, 37044 Tours, France; d.moussata@chu-tours.fr; 16 Université Paris Descartes, 75270 Paris, France; 17 UCGI Group, R&D UNICANCER, 75654 Paris, France; t-stanbury@unicancer.fr; 18 Biometrics Unit, ICM Regional Cancer Institute of Montpellier, 34298 Montpellier, France; simon.thezenas@icm.unicancer.fr; 19 Department of Surgical Oncology, Institut Curie, PSL Research University, 75005 Paris, France; pascale.mariani@curie.fr

**Keywords:** circulating tumor cells, circulating tumor DNA, liquid biopsy, metastatic colorectal cancer, FOLFIRINOX

## Abstract

The management of patients with colorectal cancer (CRC) and potentially resectable liver metastases (LM) requires quick assessment of mutational status and of response to pre-operative systemic therapy. In a prospective phase II trial (NCT01442935), we investigated the clinical validity of circulating tumor cell (CTC) and circulating tumor DNA (ctDNA) detection. CRC patients with potentially resectable LM were treated with first-line triplet or doublet chemotherapy combined with targeted therapy. CTC (Cellsearch^®^) and Kirsten RAt Sarcoma (KRAS) ctDNA (droplet digital polymerase chain reaction (PCR)) levels were assessed at inclusion, after 4 weeks of therapy and before LM surgery. 153 patients were enrolled. The proportion of patients with high CTC counts (≥3 CTC/7.5mL) decreased during therapy: 19% (25/132) at baseline, 3% (3/108) at week 4 and 0/57 before surgery. ctDNA detection sensitivity at baseline was 91% (N=42/46) and also decreased during treatment. Interestingly, persistently detectable KRAS ctDNA (*p* = 0.01) at 4 weeks was associated with a lower R0/R1 LM resection rate. Among patients who had a R0/R1 LM resection, those with detectable ctDNA levels before liver surgery had a shorter overall survival (*p* < 0.001). In CRC patients with limited metastatic spread, ctDNA could be used as liquid biopsy tool. Therefore, ctDNA detection could help to select patients eligible for LM resection.

## 1. Introduction

While most patients diagnosed with metastatic colorectal cancer (CRC) have unresectable metastases [1], some can benefit from liver surgery after conversion of unresectable disease to resectable disease by chemotherapy and targeted therapy [1,2]. In this regard, triplet chemotherapy (FOLFOXIRI) may improve the metastasis resection rate and overall survival (OS) [3]. The PRODIGE-14 trial (NCT01442935) was a randomized phase II trial intended to compare prospectively the efficacy of first-line triplet (FOLFIRINOX) versus doublet chemotherapy (FOLFOX: fluorouracil, leucovorin and oxaliplatin or FOLFIRI: fluorouracil, leucovorin and irinotecan), combined with a targeted therapy (bevacizumab in RAt Sarcoma (RAS)-mutated tumors, cetuximab in RAS wild-type tumors), in CRC patients diagnosed with potentially resectable liver metastases (LM). Results of this study have been reported elsewhere [4].

In metastatic CRC, Circulating Tumor Cell (CTC) count by the CellSearch^®^ system is known to be an independent prognostic factor in large studies, using a threshold of ≥3 CTC/7.5 mL of blood [5,6,7]; these findings were confirmed in a meta-analysis including heterogeneous detection techniques [8] and similar results were reported in other gastro-intestinal cancers [9,10]. Moreover, dynamic changes of CTC levels have been shown to be associated with progression-free survival (PFS) and OS: metastatic CRC patients with persistently elevated CTC levels after one month of chemotherapy had shorter PFS and OS than patients with decreasing CTC counts (PFS: 1.6 vs 6.2 months, p = 0.02; OS: 3.7 vs 11.0, p = 0.0002) [5].

Similarly, circulating tumor DNA (ctDNA) has proven to be useful for theranostic detection of tumor mutations [11]. While ctDNA analysis has been approved for epidermal growth factor receptor (EGFR) mutation detection in metastatic non-small cell lung cancer [12], it has been suggested as a tool for liquid biopsy in CRC. Preliminary studies addressed the overall concordance between archived tumor tissue and liquid biopsy at any stage of the metastatic disease [13]; more recent results strongly suggested that KRAS mutant subclones may be selected during anti-EGFR therapy, decreasing the overall concordance between nominal (archived tumor tissue-based) and the actual (liquid biopsy-based) KRAS status [14,15]. Furthermore, in RAS-mutated tumors, the RAS mutation may not be detectable in the plasma at first, but may later become detectable under anti-EGFR treatment [16]. In addition to these liquid biopsy applications, ctDNA levels could possibly monitor tumor dynamics [17] with early changes in ctDNA during chemotherapy in CRC associated with tumor response [18]. Detection of a residual disease by ctDNA after surgery in stage II CRC was also associated with early recurrences and poor outcome [19].

While the above-mentioned results were mostly obtained in metastatic CRC patients with heterogeneous clinical settings, we investigated the clinical validity of CTC and ctDNA detection specifically in CRC patients diagnosed with potentially resectable LM and included in the PRODIGE-14 trial. We observed a decrease of CTC and ctDNA detection rates during systemic therapy. We confirmed the prognostic value of CTC detection at baseline and during treatment, and showed that ctDNA detection was associated with a lower R0/R1 LM resection rate.

## 2. Materials and Methods

### 2.1. Patients and Treatment

The main trial, identified as NCT01442935, and its ancillary study on circulating tumor biomarkers were approved by a French ethics committee (Comité de Protection des Personnes). All subjects gave their informed consent for inclusion before they participated in the study. The study was conducted in accordance with the Declaration of Helsinki. The patients could accept to participate in the main trial but refuse the ancillary study.

The main inclusion criterion was histologically proven CRC with LM ineligible for curative resection at inclusion and without metastatic spread to other sites (except for up to 3 resectable pulmonary metastases). Other inclusion criteria were: having provided informed consent, good performance status (0–1), known exon 2 KRAS mutational status (as determined locally by standard routine technique on tumor tissue; the clinical trial was later amended to account for other KRAS and Neuroblastoma RAt Sarcoma (NRAS) mutations), adequate hematological, kidney, and liver functions, and no prior therapy for LM. Patients were randomized to either triplet (FOLFIRINOX) [20] or standard doublet (FOLFOX or FOLFIRI) chemotherapy regimens. Chemotherapy was administered in combination with cetuximab in patients with RAS wild-type cancers or with bevacizumab in patients with RAS-mutated cancers. 

The main trial objective was to demonstrate the superiority of triplet chemotherapy over doublet chemotherapy in terms of complete (R0/R1) surgical resection of LM and has already been reported [4]. A R0 resection was defined as a microscopically margin-negative resection with a distance between the margins and the tumor ≥1 mm. A R1 resection indicates a macroscopically margin-negative resection but with a distance between the margins and the tumor < 1mm. The R0/R1 resection rate was defined as the number of patients who underwent R0/R1 resection divided by the total number of patients included (R0/R1 resection, R2 resection, or no LM surgery).

### 2.2. Circulating Tumor Biomarker Detection

Three blood draws were required for this ancillary study: before starting treatment, after 1 month of systemic therapy (all patients), and before any surgical resection of LM (in patients referred to surgery after the shrinkage of LM). Blood samples were sent, within 24h, to a central laboratory (Institut Curie, Paris, France).

CTC counts were performed by experienced readers in 7.5 mL of blood (collected in CellSave^®^ tubes) using the CellSearch^®^ system (Menarini Silicon Biosystems), which has previously been reported [21]. The use of different CTC positivity thresholds was planned in order to compare the classical threshold of ≥3 CTC [5] to other thresholds and find the optimal cutoff.

For ctDNA analysis, 4 mL of plasma was thawed and cell-free DNA (cfDNA) extracted using the QIAamp^®^ Circulating Nucleic Acid Kit (Qiagen^®^), after two centrifugations as per routine procedures [22,23]. According to the manufacturer’s protocol, digital droplet PCR (ddPCR) reactions were prepared using commercially available primers and TaqMan^®^ probes (Bio-Rad^®^) with 10 ng of cfDNA. ddPCR mastermix solutions (20 µL) were transferred to a DG8 droplet generator cassette (Bio-Rad^®^) with 70 µL of oil. Emulsified PCR reactions were then transferred to a 96-well PCR plate and run on a C1000 thermal cycler (Bio-Rad^®^). Plates were analyzed on a QX-100 droplet reader (Bio-Rad^®^) with the QuantaSoft v1.7.4 software. Positivity threshold was defined as per manufacturer’s instructions, ensuring 0.1% sensitivity. Samples with a variant allele frequency <0.1% were classified as ctDNA-negative. Negative controls were used to minimize the risk of false positive. The assay could detect the G12S, G12R, G12C, G12D, G12A, G12V, and G13D mutations.

In the PRODIGE-14 trial, patients were allocated targeted therapies (cetuximab or bevacizumab) based on the KRAS exon 2 mutational analysis by local assessment on tumor tissue. While extended KRAS exon 3 and 4 and NRAS screening became mandatory in the course of the PRODIGE 14 trial, we confined our ctDNA analysis to KRAS exon 2 mutations. After one month of systemic therapy and before any surgical resection of LM, the ctDNA detection assay was only performed in patients with a known exon 2 KRAS mutation in a tumor tissue sample.

### 2.3. Statistical Analyses

The main objective of this study was to evaluate CTC and ctDNA detection rates at each time point in mCRC patients. The proportion of patients with detectable ctDNA (using the KRAS exon 2 mutation assay in cfDNA) and with detectable CTC was assessed at baseline, after one month of therapy and before LM surgery, if any. Secondary objectives were to assess the associations of circulating tumor biomarkers and baseline patient characteristics with R0/R1 LM resection and OS. Prespecified analyses were planned accordingly. Analyses conducted with the ctDNA variable (binary: detected or not detected) were also conducted with ctDNA concentration (as a continuous variable: number of mutant KRAS (KRASmut) copies per milliliter), but only for patients with KRAS exon 2 mutated tumors, as determined by routine local assessment on tumor tissues. This hypothesis-generating study had no prespecified power because the detection of circulating tumor biomarkers was done whenever possible, in patients who agreed to participate in the ancillary study. Circulating tumor biomarker detections were blinded to patients and clinicians. Patient characteristics and outcomes were prospectively collected in case report forms for all patients included in the PRODIGE-14 study. OS was defined as time from inclusion to death from any cause. Differences between categorical variables were analyzed by a chi2 test or Fisher’s exact test. Continuous variables were analyzed by a Kruskal–Wallis test. Survival curves were plotted according to the Kaplan–Meier method. Statistical significance between survival curves was assessed using the logrank test. Multivariate analysis was done by the Cox proportional hazards model with prognostic factors with a p-value of ≤0.10 in univariate analysis. Patients with one or more missing covariable were not included in the multivariate analysis. For all analyses, a p-value of ≤0.05 was considered to be statistically significant. This report was written in accordance with the REporting of tumor MARKer studies guidelines.

## 3. Results

### 3.1. Patient Characteristics

Between February 2011 and April 2015, 153 patients were enrolled. Patients characteristics are displayed in Table 1. At time of data analysis (01/2017), median follow-up was 37.2 months (IC95% (34–39); range 0–55.3 months); 96 patients were referred to surgery after undergoing a blood draw for circulating biomarker analysis, 91 patients (59%) had a R0/R1 LM resection after chemotherapy and targeted therapy, while 65 deaths (42%) had occurred.

### 3.2. CTC Detection: Correlation with R0/R1 Resection and Outcome

**Figure 1:**
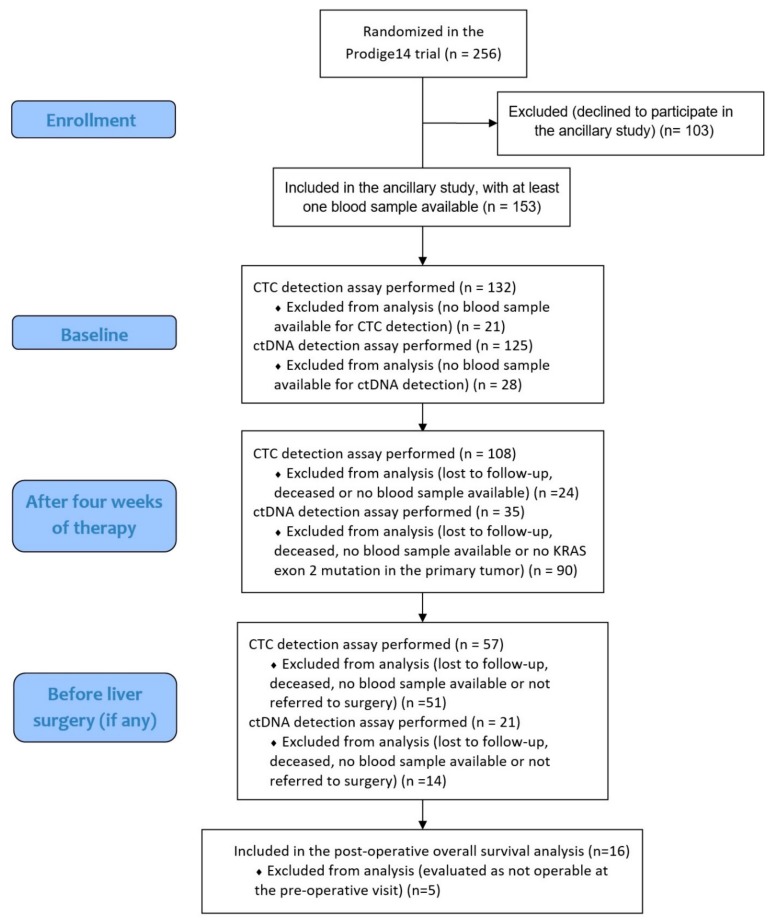
Flow chart of patients included in the analyses at the different time points.

**Figure 2:**
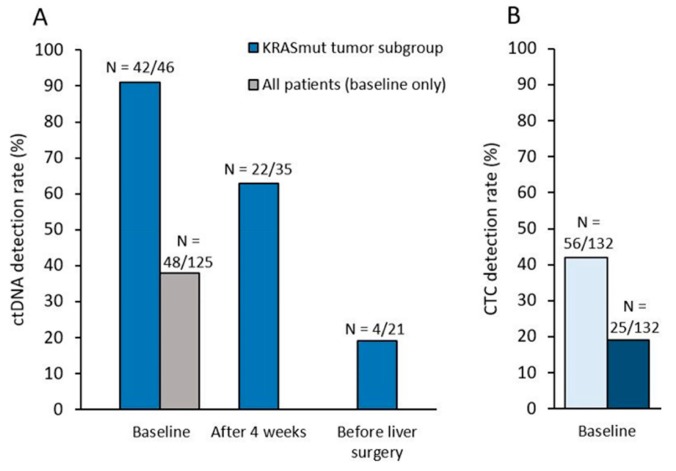
(**A**) ctDNA detection rate (KRAS exon 2 mutation with a variant allele frequency ≥ 0.1%) in all patients at baseline, and in the subgroup of patients with a KRAS exon 2 mutation as determined by routine local assessment on tumor tissues, at baseline, after 4 weeks and before liver surgery (if any). (**B**) CTC detection rate at each timepoint, with the ≥1CTC or the ≥3CTC/7.5mL of blood.

At baseline, blood samples from 132 patients were available for CTC detection (Figure 1, Supplementary Table S1). ≥1 CTC was detected in 7.5 mL of blood in 42% (N=56/132) of patients at baseline and associated with the percentage of liver infiltrated by metastases at baseline (*p* = 0.003). Using the validated ≥3 CTC/7.5 mL threshold, elevated CTC counts were observed in 19% (N = 25/132) of patients (Figure 2), and associated with the percentage of liver infiltrated by metastases at baseline (*p* = 0.001) and the synchronicity of LM (p = 0.04). CTC detection at baseline (≥1 or ≥3 CTC) was not associated with the trial’s main objective, the R0/R1 resection of LM (*p* = 0.37 and *p* = 0.18). Associations of CTCs (≥3 CTC) or ctDNA detection with baseline clinicopathological characteristics of patients are displayed in Supplementary Tables S2 and S3, respectively.

At 4 weeks, 108 patients were analyzed for CTC detection. ≥1 and ≥3 CTC were detected in 11% (N = 12/108) and 3% (N = 3/108) of patients, respectively. CTC counts decreased significantly during therapy (p < 0.0001), this decrease being similar in the treatment arms (doublet versus triplet, p = 0.98). CTC detection at 4 weeks (≥1 or ≥3 CTC) was not significantly associated with the eventual R0/R1 resection of LM, although none of the 3 patients with ≥3 CTC achieved a R0/R1 resection (p = 0.06).

Among patients referred to liver surgery, 57 patients were analyzed for CTC detection. In this selected population, ≥1 CTC was detected in 7% (N = 4/57) of patients and no patient had ≥3 CTC detected. CTC detection before surgery was not associated with R0/R1 resection (*p* = 0.37).

**Figure 3:**
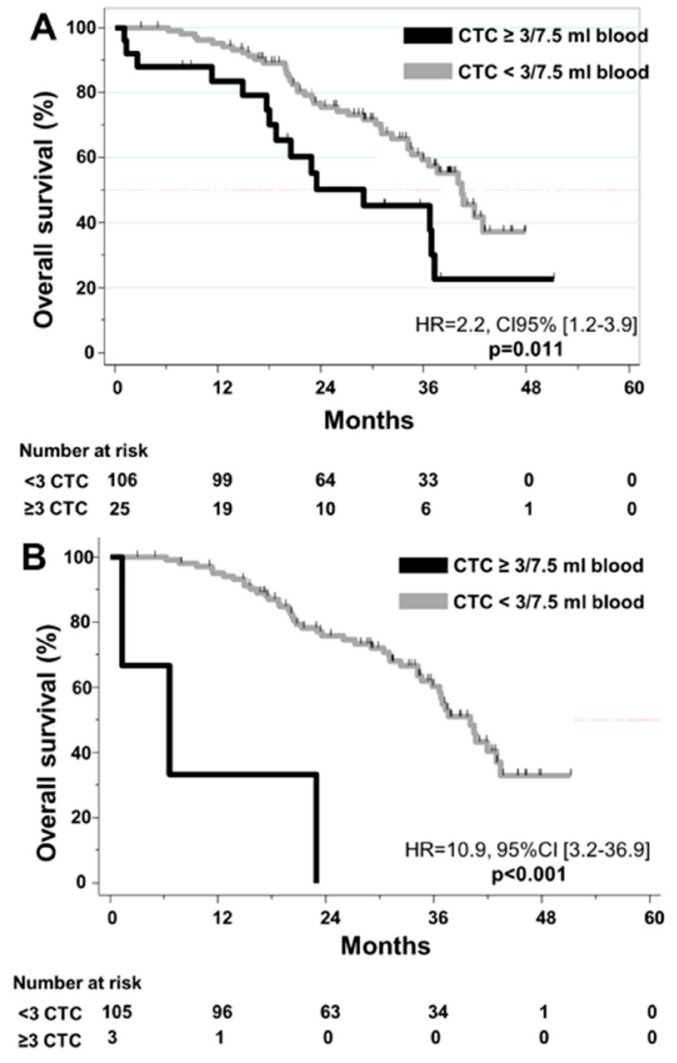
Kaplan–Meier curves for Overall Survival according to CTC detection (**A**) at baseline. (**B**) at 4 weeks.

In regards to the prognostic impact of CTC, ≥3 CTC at baseline (HR = 2.2, CI95% [1.2;3.9], p = 0.01) and at 4 weeks (HR = 10.9, 95%CI [3.2;36.9]; p < 0.001) were correlated with shorter OS (Figure 3). ≥1 CTC was significantly associated with shorter OS at 4 weeks (p = 0.04), but not at baseline (p = 0.38) or before liver surgery (p = 0.71). In multivariate analysis, ≥3 CTC was found to be an independent prognostic factor for OS at both baseline and at 4 weeks (Supplementary Table S4).

### 3.3. KRAS Mutation: Correlation between Liquid and Solid Biopsy

At baseline, blood samples from 125 patients were available for KRAS exon 2 status assessment on plasma as part of our study; 46 of these 125 patients had a KRAS exon 2 mutated tumor according to their medical files (i.e., determined by routine local assessment; Table S1). Among these 46 patients, KRASmut ctDNA was detected at baseline in 42 patients (sensitivity of the liquid biopsy = 0.91, 95%CI [0.79;0.96]). The median number of KRASmut copies/mL plasma in all 46 patients was 378 (range [0;25380]). Among the 79 patients with KRAS wild-type tumors per local assessment, 6 patients (8%) had detectable KRASmut ctDNA (nominal specificity=92%, 95%CI [0.84;0.96]). However, all 6 patients displayed high levels of ctDNA (>150 KRASmut copies/mL plasma), suggesting the actual presence of a KRAS mutation rather than a lack of specificity of the liquid biopsy. 

### 3.4. Dynamic Changes of ctDNA Levels, Correlation with R0/R1 Resection and Outcome

The following analyses were performed in the subgroup of patients with KRAS exon 2 mutated tumors, as determined by routine local assessment on tumor tissues (except at baseline for ctDNA detection as a dichotomized variable, because all patients underwent the ctDNA detection assay at this timepoint).

**Figure 4:**
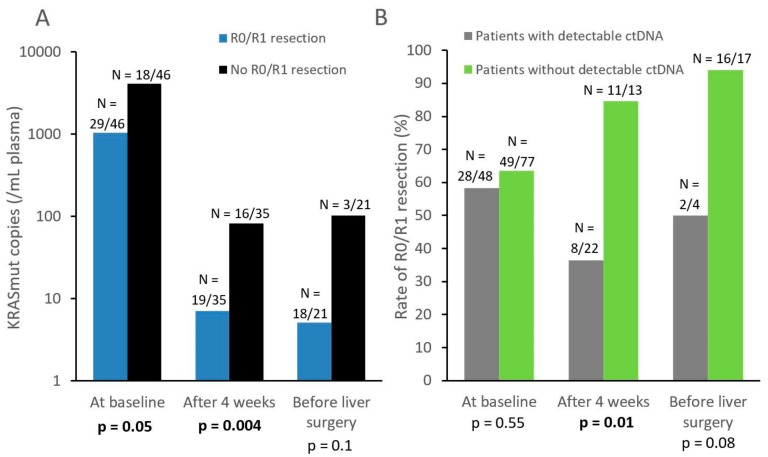
(**A**) Mean number of KRASmut copies per mL of plasma (continuous variable) at baseline, after four weeks, and before LM resection. N indicates the number of patients who achieved or did not achieve R0/R1 resection, among patients (with a KRAS mutated tumor) available for KRASmut assessment at each time point. (**B**) Rate of R0/R1 resection for patients with or without detectable ctDNA (dichotomized variable). N indicates the number of patients who achieved R0/R1 resection according to their ctDNA detection status, among patients who underwent the ctDNA detection assay at each timepoint.

At baseline, we found that KRASmut copies in plasma (continuous variable) were significantly associated with CTC positivity (≥1 CTC; Kruskal–Wallis test, p = 0.04) but not with serum markers (CA19.9: p = 0.24; CEA: p = 0.25). Baseline ctDNA concentration was however correlated with a lower R0/R1 resection rate (p = 0.05, Figure 4). 

KRAS ctDNA levels significantly decreased during therapy (p = 0.0001). 63% (N = 22/35) of patients with KRAS mutated tumors displayed detectable ctDNA at 4 weeks, while this ctDNA positivity rate dropped to 19% (N = 4/21) before surgery (Figure 2). At 4 weeks, lower ctDNA levels (as a continuous variable) were significantly correlated with eventual R0/R1 resection (p = 0.004, Figure 4). Similar results were observed with ctDNA detection as a dichotomized variable: patients with still detectable ctDNA after 4 weeks of systemic therapy had a lower R0/R1 resection rate than those with no ctDNA detected (36% vs 85%, p = 0.01, Figure 4).

**Figure 5:**
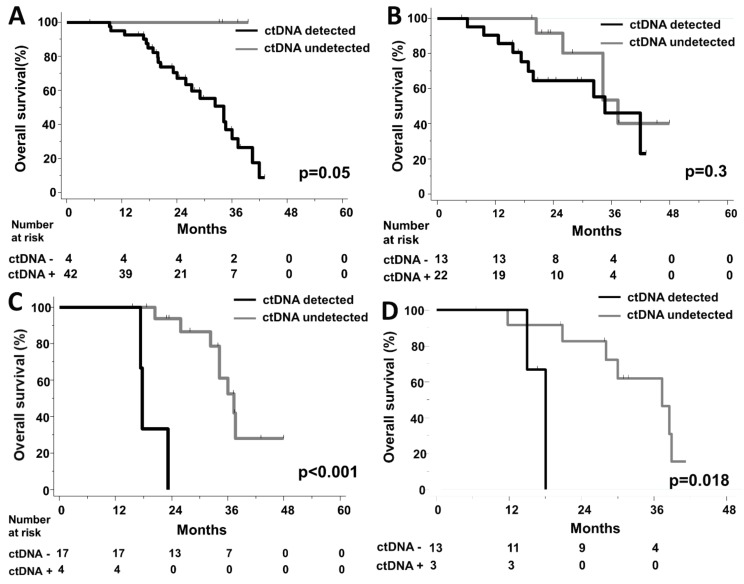
Kaplan–Meier curves for Overall Survival according to ctDNA detection (**A**) at baseline, (**B**) at 4 weeks, (**C**) before liver surgery (**D**) Kaplan–Meier curve for post-operative Overall Survival according to ctDNA detection before liver surgery.

In terms of OS, the 4 patients with no detectable ctDNA levels at baseline had an excellent prognosis (p = 0.05, HR not available; Figure 5A). ctDNA detection at 4 weeks had no prognostic impact (p = 0.31, Figure 5B, Supplementary Figure S1). However, among patients referred to LM resection, the detection of residual ctDNA levels before surgery was significantly associated with a short OS (HR = 31 CI95% [3.2;317], p < 0.001) (Figure 5C). A similar association was found with a short post-operative OS (Figure 5D).

## 4. Discussion

This is the first study to investigate the clinical validity of both CTC and ctDNA in patients with potentially resectable LM of CRC in a prospective clinical trial. These patients should be treated with intensive first-line systemic therapy combining poly-chemotherapy and the most appropriate targeted therapy (anti-EGFR antibodies in RAS wild-type tumors). Our study found that before the start of systemic therapy, the results of the assessment of tumor mutation status using ctDNA were closely correlated with those of local testing. Even more interestingly, we identified a few patients considered KRAS wild-type by tumor tissue sequencing that had significant KRAS mutant levels in their blood. A similar discrepancy was observed, but at much higher rates, in studies that focused on heavily pre-treated patients [15], suggesting a role of prior anti-EGFR therapies in the emergence of KRAS mutants subclones. Importantly, the probable benefit of anti-EGFR therapy is very limited in such cases [14]. In chemotherapy-naïve patients, the recent RAS Mutation Testing in the Circulating Blood of Patients With Metastatic Colorectal Cancer (RASANC) study [24] found that 8 of 412 patients had a RAS mutation in plasma but not in the primary tumor by local assessment. However, the authors performed central re-analysis on 6 of 8 tumor samples, by next-generation sequencing (NGS) or ddPCR, and found RAS mutations in all six samples. These results, and the shorter testing time, strongly suggest that ctDNA analysis might become a valuable theranostic tool in patients diagnosed with potentially resectable LM.

In addition to liquid biopsy applications at baseline, the clinical validity of ctDNA quantification was investigated at different time points during therapy. First, systemic therapy induced a significant decrease in ctDNA levels, highlighting that liquid biopsy has a very limited sensitivity once therapy has been initiated. We also found that ctDNA levels at different time points yielded significant prognostic information: undetectable ctDNA levels at baseline tended to be a prognostic factor, as demonstrated in other cancers such as metastatic lung cancer [25], ctDNA being correlated with tumor burden in various cancer types [26]. More interestingly, the absence of ctDNA at 4 weeks was correlated with a very high R0/R1 resection rate of LM (85%), suggesting that this biomarker could help decide whether liver surgery is appropriate for patients.

Finally, in patients referred to surgery for LM resection, persistently detectable ctDNA levels before surgery was associated with short post-surgical OS, suggesting that LM were not fully responding to therapy and/or that extra-hepatic micro-metastases were present. A study by Narayan and colleagues [27] in 59 metastatic CRC patients who underwent LM resection found an association between worse disease-specific survival and the detection of circulating mutant *TP53* copies during surgery (but not with ctDNA). However, blood samples were only obtained during and after surgery. Recent studies have found an association between dynamic changes in ctDNA detection and outcome in CRC, in the adjuvant setting, or in the metastatic setting. In the adjuvant setting [28], change of ctDNA status (as a dichotomized variable: detected or not detected) from positive to negative or from negative to positive was associated with respectively superior or lower recurrence-free survival. In the metastatic setting, a recent study [29], using a composite marker evaluating the decrease of ctDNA levels during chemotherapy, demonstrated that it could be used to predict response, progression-free survival, and OS.

If confirmed by further studies, we hypothesize that the absence of detectable ctDNA might become an important criterion prior to any LM resection in this patient population.

Regarding ctDNA analyses, limitations of our study include the limited number of KRAS mutated tumors enrolled and the focus on KRAS exon 2 mutations, as predefined in the study protocol at time of initiation, with no assessment of other KRAS, NRAS, and Rapidly Accelerated Fibrosarcoma homolog B (BRAF) mutations. Of note, while assessing several mutation hotspots in a single assay is usually achieved by NGS; multiplex ddPCR [30] and, more recently, drop-off ddPCR [31] may allow the screening of several hotspots in a single reaction. Larger mutation panel or methylation patterns can be used to detect and quantify ctDNA in a larger proportion of patients [18,24,32]. In the RASANC study [24], plasma samples from chemotherapy-naïve metastatic CRC patients were analyzed by NGS combined with methylation ddPCR, which allowed for a high detection rate of ctDNA (329/425, 77%).

Regarding CTC detection, our study showed its correlation with ctDNA levels, as already reported in patients with uveal melanoma LM [33]. However, the CTC detection rate was lower in our patient population than in prior studies in non-resectable metastatic CRC patients [5,7], probably because of the limited tumor burden in patients included in this study. While our study confirmed the prognostic impact of CTC count at baseline, the number of patients with persistently elevated CTC counts during therapy appeared very limited and prevents any clinical utility in this clinical setting, despite a proven clinical validity. We propose that more sensitive CTC detection techniques [34] be investigated in metastatic CRC to assess the clinical utility of CTC level, such as those relying on microfluidics [35], on EpCAM-independent CTC detection [36] and/or on the screening of larger blood volume [37].

Lastly, newly developed circulating biomarkers such as free serum amino acids [38] could be compared to CTC or ctDNA detection for their prognostic value. Similarly, circulating extracellular matrix components have been evaluated as biomarkers for cancer diagnosis and prognosis in various tumor types [39].

This prospective study showed that CTC and ctDNA had different detection profiles in mCRC patients with potentially resectable LM, the latter demonstrating interesting validity with regards to liquid biopsy and pre-operative prognostic applications.

## Figures and Tables

**Table 1:** Patients characteristics. N = 153 patients included in the study.

Characteristics	Median Value or Number of Patients
**Age**, years	Median: 60
	Range: 25–75
**Performance Status**	
0	95 (63%)
1	57 (37%)
**Prior resection of the primary tumor**	
No	103 (67%)
Yes	50 (33%)
**Synchronous liver metastases**	
No	19 (12%)
Yes	134 (88%)
**% of liver infiltrated by metastases**	
0–25%	41 (45%)
26–50%	28 (30%)
51–75%	15 (16%)
>75%	8 (9%)
**CEA**	
Normal	17 (11%)
>upper limit of normal	134 (89%)
**CA19.9**	
Normal	39 (37%)
>upper limit of normal	66 (63%)
**KRAS exon 2 mutation in tumor sample**	
No	94 (61%)
Yes	59 (39%)
**Chemotherapy**	
Doublet + targeted therapy	75 (49%)
Triplet + targeted therapy	78 (51%)
**R0/R1 resection of liver metastases**	
No	62 (41%)
Yes	91 (59%)

## Supplementary Materials

The following are available online at https://www.mdpi.com/2073-4409/8/6/516/s1, Table S1: blood samples available. Table S2: associations of CTCs (as a dichotomous variable: ≥3 or <3 CTC/7.5 mL) and clinicopathological characteristics of patients at baseline (when available), treatment arm, or primary endpoint. Table S3: associations between ctDNA detection (as a dichotomous variable) and clinicopathological characteristics of patients at baseline (when available), treatment arm, or primary endpoint. Table S4: multivariate Cox regression with CTC detection at baseline and at 4 weeks (Overall Survival). Figure S1: post-operative overall survival according to ctDNA detection at 4 weeks.
