# Supplementary material for: Circulating Tumor Cells and Circulating Tumor DNA Detection in Potentially Resectable Metastatic Colorectal Cancer: A Prospective Ancillary Study to the Unicancer Prodige-14 Trial
Source: Cells. 2019 May 28;8(6):516. doi: 10.3390/cells8060516 (PMC6627974; doi:10.3390/cells8060516)

Table S1: blood samples available.

	Biomarker assessed	
	CTC	ctDNA detection<br>(*KRAS* mutated tumors)
Baseline	All patients<br>(KRASwt and KRASmut tumors)<br>N = 132	All patients<br>(KRASwt and KRASmut tumors)<br>N=125 (*)
4 weeks	All patients<br>(KRASwt and KRASmut tumors)<br>N=108	Patients with KRASmut tumors<br>N=35
Before LM surgery	All patients<br>(KRASwt and KRASmut tumors)<br>N=57	Patients with KRASmut tumors<br>N=21

(*) of whom N=46 patients with KRASmut tumors

Table S2: associations between CTC levels (as a dichotomous variable: ≥3 or <3 CTC/7.5 mL) and clinicopathological characteristics of patients at baseline (when available), treatment arm or primary endpoint.

Characteristics	<3 CTC/7.5 mL	≥3 CTC/7.5 mL	p (Fisher's exact test)
**Performance Status**			
0	66 (62%)	15 (60%)	0.82
1	40 (38%)	10 (40%)	
**Prior resection of the primary tumor**			
No	73 (68%)	19 (76%)	0.63
Yes	34 (32%)	6 (24%)	
**Synchronous liver metastases**			
No	16 (15%)	0 (0%)	0.04
Yes	91 (85%)	25 (100%)	
**% of liver infiltrated by metastases**			
00-25%	33 (52%)	2 (13%)	0.001
26-50%	18 (29%)	5 (31%)	
51-75%	10 (16%)	4 (25%)	
>75%	2 (3%)	5 (31%)	
**Lung metastases**			
No	95 (90%)	22 (92%)	1.0
Yes	10 (10%)	2 (8%)	
**CEA**			
Normal	11 (10%)	3 (12%)	0.73
> upper limit of normal	94 (90%)	22 (88%)	
**CA19.9**			
Normal	31 (41%)	2 (12%)	0.04
> upper limit of normal	44 (59%)	14 (88%)	
**KRAS exon 2 mutation in tumor sample**			
No	61 (57%)	20 (80%)	0.04
Yes	46 (43%)	5 (20%)	
**Chemotherapy**			
Doublet + targeted therapy	52 (49%)	10 (40%)	0.51
Triplet + targeted therapy	55 (51%)	15 (60%)	
**R0/R1 resection of liver metastases**			
No	40 (37%)	13 (52%)	0.18
Yes	67 (63%)	12 (48%)	

Table S3: associations between ctDNA detection (as a dichotomous variable) and clinicopathological characteristics of patients at baseline (when available), treatment arm or primary endpoint.

Characteristics	ctDNA not detected	ctDNA detected	p (Fisher's exact test)
**Performance Status**			
0	44 (57%)	33 (70%)	0.18
1	33 (43%)	14 (30%)	
**Prior resection of the primary tumor**			
No	55 (71%)	33 (69%)	0.84
Yes	22 (29%)	15 (31%)	
**Synchronous liver metastases**			
No	10 (13%)	5 (10%)	0.78
Yes	67 (87%)	43 (90%)	
**% of liver infiltrated by metastases**			
00-25%	20 (42%)	12 (44%)	0.95
26-50%	14 (29%)	8 (30%)	
51-75%	8 (17%)	5 (19%)	
>75%	6 (12%)	2 (7%)	
**Lung metastases**			
No	67 (89%)	42 (89%)	1.0
Yes	8 (11%)	5 (11%)	
**CEA**			
Normal	6 (8%)	6 (13%)	0.53
> upper limit of normal	70 (92%)	41 (87%)	
**CA19.9**			
Normal	22 (43%)	9 (25%)	0.11
> upper limit of normal	29 (57%)	27 (75%)	
**KRAS exon 2 mutation in tumor sample**			
No	73 (95%)	6 (12%)	0.001
Yes	4 (5%)	42 (88%)	
**Chemotherapy**			
Doublet + targeted therapy	38 (49%)	24 (50%)	1.0
Triplet + targeted therapy	39 (51%)	24 (50%)	
**R0/R1 resection of liver metastases**			
No	28 (36%)	20 (42%)	0.55
Yes	49 (64%)	28 (58%)	

Table S4: Multivariate Cox regression with CTC detection at baseline and at 4 weeks (Overall Survival). Number of patients included in the multivariate analysis: N = 91 at baseline, N = 75 at 4 weeks. Patients with one or more missing covariable were not included in the multivariate analysis.

	**Baseline**		**At 4 weeks**	
**Factors**	**HR (95% CI)**	**p**	**HR (95%CI)**	**p**
Chemotherapy: triplet versus doublet	0.42 [0.2;0.9]	0.019	0.47 [0.2;1.1]	0.083
KRAS status: mutated versus wild type	3.9 [1.6;9.6]	0.003	3.4 [1.3;8.9]	0.013
CEA: elevated versus normal	2.7 [1.1;6.1]	0.02	2.2 [0.95;5.2]	0.065
CTC count: ≥3/7.5 mL versus <3	2.8 [1.3;6]	0.008	34.6 [4.5;266]	0.001

Figure S1: post-operative overall survival according to ctDNA detection at 4 weeks.

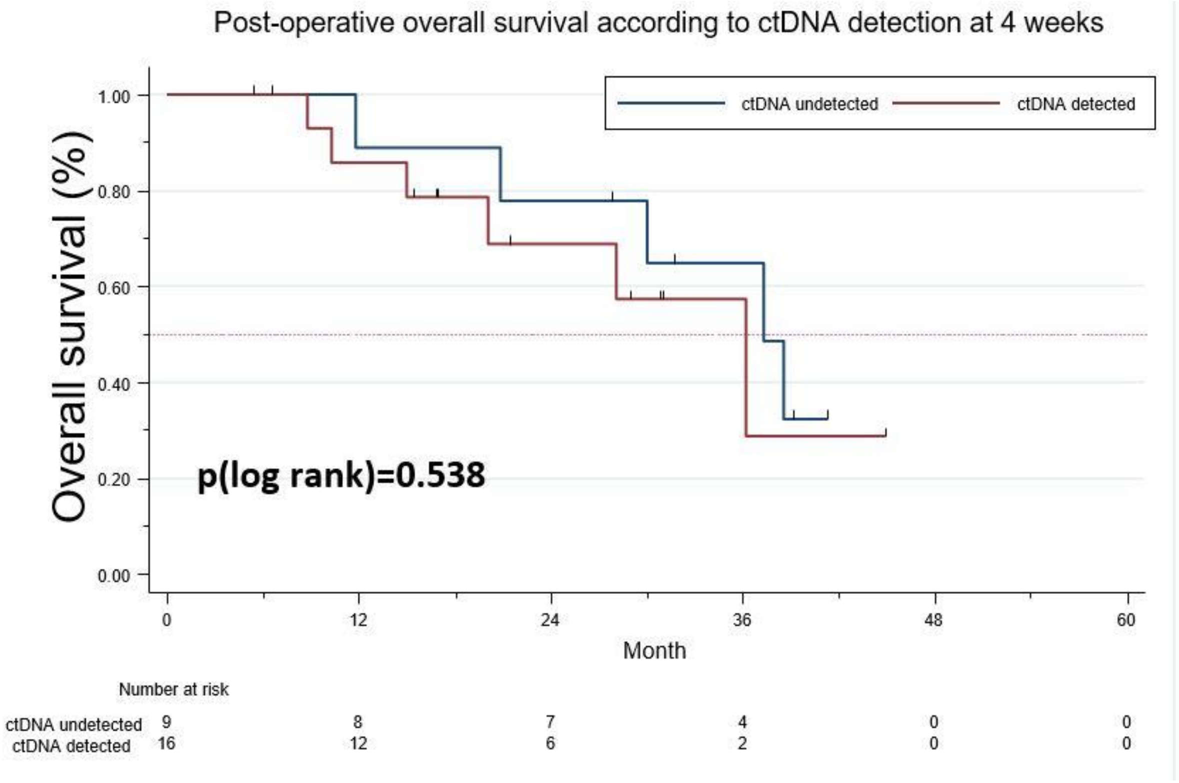

Supplement: Supplementary file 1 [file cells-08-00516-s001.pdf]
